# Changes in pollinator fauna affect altitudinal variation of floral size in a bumblebee-pollinated herb

**DOI:** 10.1002/ece3.1191

**Published:** 2014-08-18

**Authors:** Yusuke Nagano, Kota Abe, Tomoaki Kitazawa, Mitsuru Hattori, Akira S Hirao, Takao Itino

**Affiliations:** 1 Department of Biology, Faculty of Science, Shinshu University3-1-1 Asahi, Matsumoto, Nagano, 390-8621, Japan; 2 Sugadaira Montane Research Center, University of TsukubaUeda, Nagano, 386–2201, Japan; 3 Institute of Mountain Science, Shinshu University3-1-1 Asahi, Matsumoto, Nagano, 390-8621, Japan

**Keywords:** Geographic selection mosaic, local adaptation, mechanical fit, phenotypic selection, pollination efficiency

## Abstract

Geographic trait variations are often caused by locally different selection regimes. As a steep environmental cline along altitude strongly influences adaptive traits, mountain ecosystems are ideal for exploring adaptive differentiation over short distances. We investigated altitudinal floral size variation of *Campanula punctata* var. *hondoensis* in 12 populations in three mountain regions of central Japan to test whether the altitudinal floral size variation was correlated with the size of the local bumblebee pollinator and to assess whether floral size was selected for by pollinator size. We found apparent geographic variations in pollinator assemblages along altitude, which consequently produced a geographic change in pollinator size. Similarly, we found altitudinal changes in floral size, which proved to be correlated with the local pollinator size, but not with altitude itself. Furthermore, pollen removal from flower styles onto bees (plant's male fitness) was strongly influenced by the size match between flower style length and pollinator mouthpart length. These results strongly suggest that *C. punctata* floral size is under pollinator-mediated selection and that a geographic mosaic of locally adapted *C. punctata* exists at fine spatial scale.

## Introduction

Trait variations across a wide geographic range (e.g., along latitude) are greatly influenced by isolation caused by distance or a geographic barrier (Gould and Johnston 1972). In contrast, those across a narrow geographic range (e.g., along altitude) are often caused by locally different selection regimes (Endler 1977; Olsson and Agren 2002; Herrera et al. 2006; Anderson and Johnson 2007; Toju 2008) and are influenced by the balance between gene flow and selection strength (Saint-Laurent et al. 2003; Kawecki and Ebert 2004; Räsänen and Hendry 2008). Such trait variations across a narrow range are often easily homogenized by gene flow, causing them to be obliterated (Kawecki and Ebert 2004). Under strong local selective pressure, however, such phenotypic divergence is sustainable even in the presence of gene flow (Sambatti and Rice 2006; Byars et al. 2009; Gonzalo-Turpin and Hazard 2009).

In mountain ranges, steep environmental clines along altitude can strongly influence adaptive traits and potentially cause adaptive diversification within a narrow geographic range (Byars et al. 2007; Mila et al. 2009). Thus, mountain ecosystems are ideal for exploring adaptive differentiation over short distances. Typically, a decrease in plant vegetative size with increasing altitude is caused by clinal abiotic environmental changes (e.g., meteorological changes) (Körner 2003; Fabbro and Körner 2004; Alexander et al. 2009; Hautier et al. 2009). Altitudinal changes in biotic interactions can also indirectly influence plant traits (Dohzono and Suzuki 2010), but plant trait variations caused by such biotic interactions have seldom been explored, especially from an evolutionary perspective (Schemske et al. 2009).

In angiosperms, selective pressure imposed by pollinators plays an important role in floral evolution (Stebbins 1970; Herrera 1996; Harder and Johnson 2009). Therefore, if pollinator assemblages differ among populations, local adaptation of floral traits should also be observed. In fact, floral traits have been shown to change geographically in relation to differences in pollinator assemblages (Galen 1996; Herrera et al. 2006; Gómez et al. 2008; Newman et al. 2014; Sun et al. 2014). Such ecological differentiation of floral traits might promote genetic divergence among populations through pollinator isolation among populations (Stebbins 1970; Coyne and Orr 2004; Kay 2006; Nosil 2012).

Herrera et al. (2006) proposed a five-step protocol for identifying geographic differentiation in floral traits driven by spatially variable selection by pollinators, as follows: (i) document geographic variations in pollinator assemblages; (ii) demonstrate pollinator-mediated selection on floral traits; (iii) assess geographic divergence in selection; (iv) evaluate the match between divergent selection and phenotypic divergence; and (v) determine whether population differences in floral traits have a genetic basis. Although several studies have shown that altitudinal variation of floral traits is related to altitudinal differences in pollinator assemblages (step i; Malo and Baonza 2002; Herrera 2005; Nattero and Cocucci 2007; Maad et al. 2013; Newman et al. 2014; Sun et al. 2014), few studies have demonstrated steps ii–v, that is, altitudinal local adaptation of floral traits to pollinator assemblages (but see Gómez et al. 2009).

The aim of this study was to examine altitudinal floral size variation in *Campanula punctata* Lam. var. *hondoensis* (Kitam.) Ohwi, according to steps i to iv of the proposed protocol. We studied the altitudinal change in assemblages of *C. punctata* pollinators (step i) and examined whether floral size was subject to selection from locally different pollinator sizes (steps ii and iii) and the spatial correlation between local pollinator size and floral size (step iv). With regard to step v, Inoue et al. (1995) previously showed that variation in floral size in *C. punctata* has a genetic basis.

**Figure 1:**
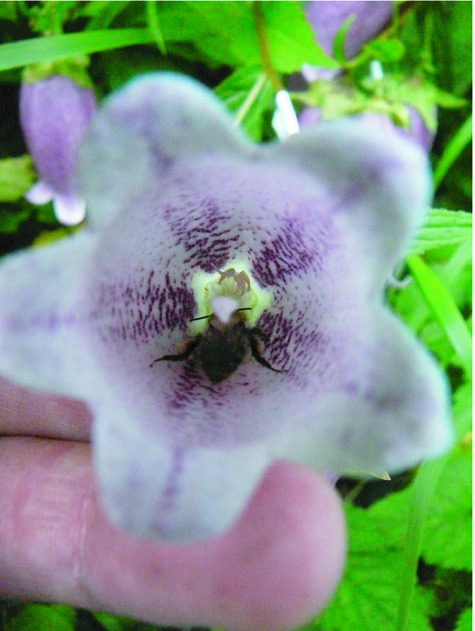
The male-phase flower of *Campanula punctata* and a visiting bumblebee. In this phase, the pollens are adhered on the lateral surface of the style. Several days later, in female phase, the tip of the style unfolds and the stigmatic lobes appear. The match between floral style length and pollinator mouthpart length is important in the plant male fitness (pollen removed by bee visits).

**Figure 2:**
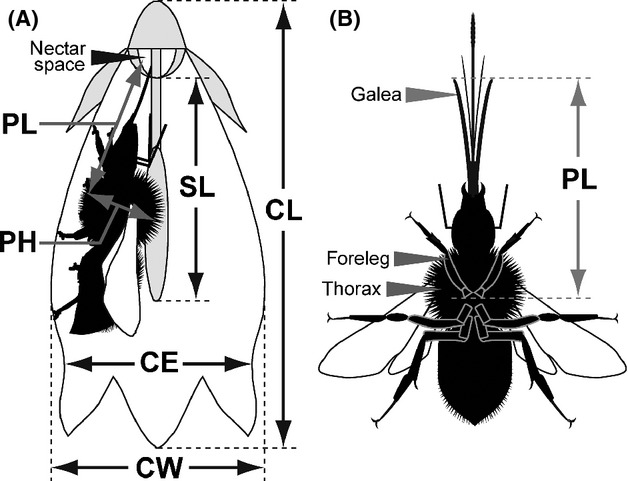
(A) A male-phase flower of *Campanula punctata* and a visiting bumblebee. In this phase, the pollen grains adhere to the lateral surface of the style. Several days later, when the flower enters the female phase, the tip of the style unfolds and the stigmatic lobes appear. CE, corolla entrance diameter; CW, corolla width; SL, style length; CL, corolla length; PL, pollinator length (distance from the base of the foreleg to the tip of the galea); PH, pollinator height (maximum thorax height). (B) Ventral side of bumblebee and definition of PL measurement.

When a bumblebee (*Bombus* spp.), the main pollinator of *C. punctata* (Inoue and Amano 1986; Kobayashi et al. 1997)*,* enters the corolla of *C. punctata* to collect nectar, the dorsal surface of its thorax just to uches the lateral surface of the style, provided that there is a good fit between pollinator and flower (Fig.1). In *C. punctata*, the style has both male and female functions (Fig.2); therefore, the style–pollinator size match is expected to greatly influence pollination efficiency in *C. punctata*. An example is provided by the miniaturized floral size of a sister species of *C. punctata*, *Campanula microdonta* Koidz., which inhabits a small island where tiny solitary bees are the pollinators (Inoue and Amano 1986; Inoue 1988), suggesting that the floral size is adapted to the size of the local pollinator. In the mountains of central Japan, where bumblebees pollinate *C. punctata,* bumblebee assemblages differ at different altitudes (Tomono and Sota 1997), suggesting that selective pressures on floral size in *C. punctata* may differ along altitude. For this reason, we considered this a suitable system in which to explore fine geographic scale adaptation of floral size in *C. punctata*, a pollinator generalist, to locally different pollinator assemblages along an altitude gradient.

In this study, we show that (1) the assemblages of *C. punctata* pollinators change along altitude; (2) altitudinal floral size variation in *C. punctata* is highly correlated with local pollinator size and not with altitude itself; and (3) the flower-pollinator size match influences plant fitness.

## Methods

### Plant species

*Campanula punctata* Lam. var. *punctata* (Campanulaceae) is a protandrous, self-incompatible perennial herb with large bell-shaped, magenta-white flowers that are pollinated primarily by bumblebees (Kobayashi et al. 1997). A variety of this species, *C. punctata* Lam. var. *hondoensis* (Kitam.) Ohwi, is distributed in central Japan, where it grows across a wide altitudinal range, in environments from coastal (0 m a.s.l.) to subalpine (about 2000 m a.s.l.). In *Campanula* flowers, pollen grains are not directly transferred from the anthers to the pollinator; rather, pollen grains are shed onto the style, while it is tightly surrounded by the anthers in the young bud. By the time the flower opens, the stamens have shriveled and the pollen adhering to the style is ready for transfer to pollinators as they crawl into the corolla (male phase; Figs.1 and 2). The stigma does not become receptive until about 2 days after the flower opens, at which time the tip of the style unfolds and the stigmatic lobes appear (female phase) (Inoue and Kawahara 1990; Kobayashi et al. 1997).

### Study area

We studied altitudinal changes in floral size and pollinator assemblages in 12 populations of *C. punctata* var. *hondoensis* in three mountain regions in central Japan (Table1) in 2010 and 2011. Four populations in the Norikura region (1475―2269 m a.s.l., Na-4, Na-5, Na-6, and Na-7) were also used to examine the effect of the flower–pollinator size match on plant fitness. We studied each population during the peak flowering season of that population. The geographic distances between the three mountain regions were 20–70 km. Within each mountain region, the geographic distances between the highest and lowest population were 21.1 km in Norikura, 7.2 km in Utsukushigahara, and 15.2 km in Yatsugatake.

**Table 1:** The 12 populations of *Campanula punctata* where the altitudinal variation in floral size and pollinator assemblages were studied.

Population	Altitude (m)	Latitude (N)	Longitude (E)	Year
Norikura				
Na-1	744	36°10′53″	137°47′46″	2010, 2011
Na-2	930	36°09′12″	137°45′13″	2010
Na-3	1470	36°06′59″	137°37′48″	2011
Na-4^1^	1475	36°07′25″	137°37′34″	2010, 2011
Na-5^1^	1663	36°07′51″	137°37′54″	2010
Na-6^1^	1945	36°06′58″	137°35′32″	2010, 2011
Na-7^1^	2269	36°07′13″	137°34′24″	2010, 2011
Yatsugatake				
Ya-1	944	36°00′05″	138°12′02″	2011
Ya-2	2103	36°03′31″	138°21′14″	2011
Utsukushigahara				
Ut-1	792	36°15′46″	138°00′00″	2011
Ut-2	1531	36°14′48″	138°03′13″	2011
Ut-3	1904	36°15′08″	138°04′43″	2011

1 Populations in which the influence of the pollinator–flower size match on plant fitness was also examined.

### Factors influencing local floral size

#### Variations in local pollinator assemblages

We determined pollinator assemblages of *C. punctata* along the altitudinal gradient. We selected the largest flower patch (size range, 20–50 m^2^) in each of the 12 populations and conducted a census, taking between 3 and 20 h for each patch, between 06:00 and 16:00 during the peak of the flowering season in the summers of 2010 and/or 2011. During each census, we counted the number of individuals of each bumblebee species visiting the flower patch. Then, we calculated the relative abundance of each species as the ratio of the number of individuals of that species to the total number of individuals of all bumblebee species visiting the patch.

**Figure 3:**
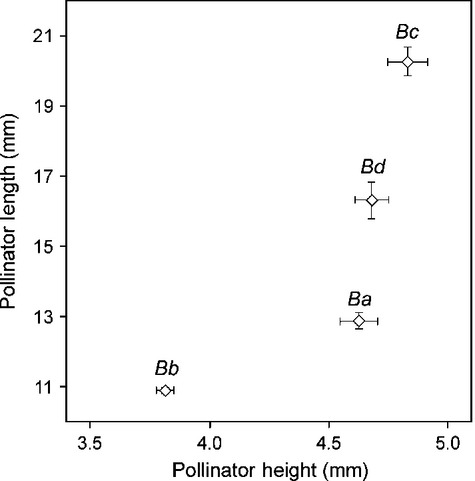
Relationship between pollinator length (PL) and pollinator height (PH) (mean ± SE) in the four dominant bumblebee species visiting *Campanula punctata*. *Bc*, *Bombus consobrinus* (worker); *Bd*, *B. diversus* (worker); *Ba*, *B. ardens* (male); *Bb*, *B. beaticola* (worker).

We randomly collected 1–41 individuals of each bumblebee species in the Norikura region. For each individual, we noted the caste and measured two body size parameters, pollinator length (PL) and pollinator height (PH) (Fig.2), with a digital caliper with a 0.01 mm precision. Here, PL is defined as the distance from the base of the foreleg to the tip of the galea (Fig.2) because bumblebees insert their galea into the nectar space where only the galea but not the glossa can be pierced and reached. PH is defined as the thorax height. The PL to style length (SL) ratio (PL:SL) and the style–pollinator room (CW/2 – PH) both may potentially affect pollen removal from the style and its deposition onto the stigma by the bees (Fig.2). To evaluate the average size of pollinators visiting each population, we calculated the pollinator size index (PSI):


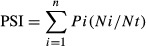


where *n* = the total number of bumblebee species visiting a flower population (patch), *Pi* = mean body size (PL or PH) of the *i*th bumblebee species (see Fig.3), *Ni* = the number of foraging bouts of the *i*th bumblebee species observed at the patch, and *Nt* = the number of foraging bouts of all bumblebee species observed at the patch (thus, *Ni*/*Nt* is the relative abundance of *i*th bumblebee species visiting the population).

#### Floral size variation

From each population, we haphazardly selected 20–60 individual plants and then 1–4 flowers from each plant for morphological measurement. For each flower, we measured four floral size parameters with a digital caliper with a 0.01 mm precision: corolla entrance width (CE), corolla width (CW), style length (SL), and corolla length (CL) (Fig.2). When more than one flower from an individual plant was measured, we used the mean value of each parameter as the data for that plant. We expected CE to constrain the size of the pollinator that can enter the corolla, and CW, used as a proxy for the space available for the bee between the style and the corolla, to affect the efficiency of pollen removal and deposition by the bees. The relationship between PL and SL should also affect pollination efficiency. We considered CL to be an indicator of each flower's display size. We performed a principal component analysis (PCA) using these four floral sizes on the pooled data of all populations and estimated the first two principal component scores (PC1 and PC2), which explained most of the variance in the four floral sizes. After the flowering season, we counted the total number of flowers (capsules) per plant (NFP). We compared NFP and floral size parameters (CE, CW, SL, CL, PC1, and PC2) among populations using one-way analysis of variance (ANOVA). For these analyses, we used R version 2.15.0 software (R Development Core Team 2012).

#### Bee preference for floral size

**Figure 4:**
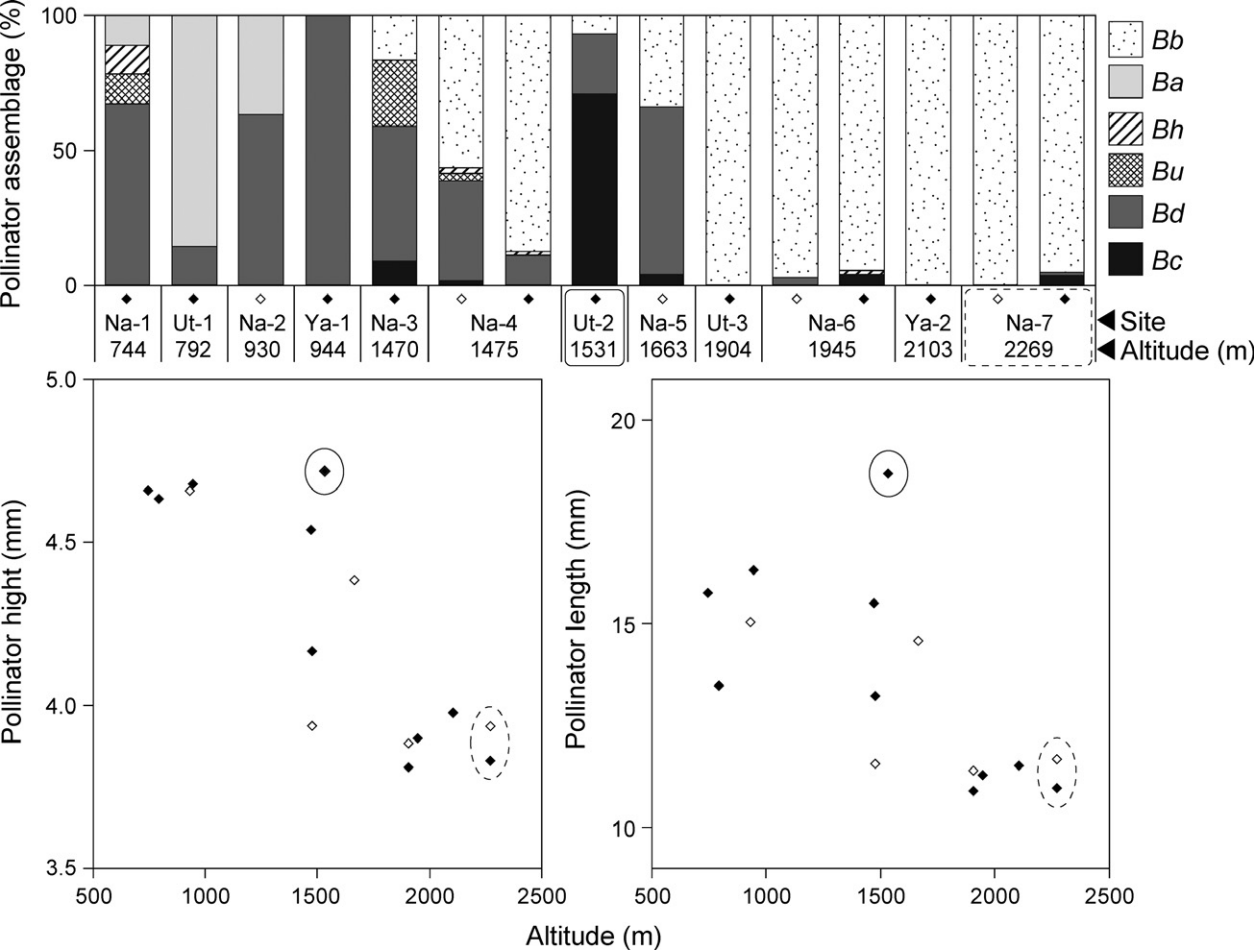
Top: Altitudinal change in assemblages of *Campanula punctata* pollinators (bumblebees). From smallest to largest: *Bb*, *B. beaticola* workers and males; *Ba*, *B. ardens* males; *Bh*, *B. honshuensis* workers; *Bu*, *B. ussuriensis* workers; *Bd*, *B. diversus* workers; *Bc*, *B. consobrinus* workers. Bottom: Altitudinal variation in pollinator height (left) or pollinator length (right) in the 12 populations. See Fig.2 for the dimensions indicated by main pollinator length and height. Each diamond represents a population (white, censused in 2010; black, censused in 2011). Populations Ut-2 and Na-7 are circled with a solid and dashed line, respectively. See Fig.5 for more information.

To examine whether bumblebee species prefer to specific floral size, we measured floral size (CL) of those flowers visited by bumblebees in Na-4 and Na-5 where two bumblebee species predominantly visited *C. punctata* flowers (large species: *B. diversus* and small species: *B. beaticola*, see Figs.3 and 4).

#### Factors influencing local floral size

To examine factors influencing floral size parameters (CE, CW, SL, CL, PC1, and PC2), we used a generalized linear mixed model (GLMM) with a Gaussian error distribution, in which the pollinator size index (PSI), total number of flowers per plant, and the altitude of each population were included as fixed effects, and year and population were random effects. We excluded four populations (Ut-1, Ut-3, Ya-2, and Na-3; see Table1) where we could not count the number of flowers from the analysis, because artificial or natural disturbance (e.g., mowing or feeding damage) had occurred in these population before counting. The analyses were performed with the nlme package of R version 2.15.0 software (R Development Core Team 2012).

### Effect of the flower–pollinator size match on pollen removal and pollen deposition

We evaluated the effects of the flower–pollinator size match on pollen removal from the style onto a bee's thorax (an estimate of male flower fitness), pollen fall (cost of a size mismatch), and pollen deposition from a bee onto the stigma (female flower fitness). To evaluate the effects of the size match on male and female fitness, we pooled the data from the different bumblebee species because their body shape (i.e., PL:PH) was relatively constant not only within species but also among species (in pooled data of all species, Pearson's correlation coefficient, *r* = 0.812, *P* < 0.001, *n* = 123) (cf., Fig.3).

#### Initial number of pollen grains on the style

First, we estimated the initial number of pollen grains (before any pollinator visit) on each style. We haphazardly selected 50 flower buds from each of four populations (Na-4, Na-5, Na-6, and Na-7) and placed a nylon mesh bag around each bud until it opened. As each flower opened, we measured the floral dimensions and collected a sample of pollen, which was dislodged by sonication of the styles for 5 min in a solution of 1.5 ml of 5% formaldehyde, 5% acetic acid, and 50% ethanol in water (FAA). Each sample was vortexed for 10 s, and then, the number of pollen grains in three subsamples of 5 *μ*L each was counted under a light microscope (×100). To estimate the total number of grains on the style, we summed those counted in the three subsamples and multiplied the total by 100 (Kobayashi et al. 1997). We then performed a linear regression of the total number of pollen grains on each style against SL (Pearson's correlation coefficient *r* = 0.42, *P* < 0.005, *n* = 50) and used the regression equation [No. pollen grains = 70.69 × SL + (1.03 × 10^5^)] to estimate the initial number of pollen grains on each style.

#### Pollen removal

We calculated the number of pollen grains that were removed from the style per bee visit as the difference between the initial pollen number and the sum of the pollen grains remaining on the style after the bee visit or fallen from the style. We haphazardly selected 49 flower buds in the field (Na-4, Na-5, Na-6, and Na-7) and placed a nylon mesh bag around each bud until it opened. We then removed the bag from each newly opened flower (male phase) to allow a bumblebee visit. The pollen grains that fell out of the flower during each visit were collected on a clean sheet of paper placed under the flower and then put into FAA until counting. We timed each bumblebee visit with a stopwatch. After the visit, we caught the bumblebee as it crawled out of the corolla, identified its species and caste, and measured its body size (PL and PW). We also measured the size of each flower (CE, CW, SL, and CL). The style was preserved in FAA until the pollen grains remaining on it were counted. The initial pollen number was then estimated from the style length as already described. We calculated the number of pollen grains removed from the style by the bee visit as follows:

No. removed grains = No. initial grains − (No. remaining grains + No. fallen grains)

The 49 flowers were visited by workers of *B. ussuriensis* (*n* = 2), *B. diversus* (*n* = 7), and *B. honshuensis* (*n* = 4), and by workers (*n* = 30) and males (*n* = 6) of *B. beaticola*.

#### Pollen deposition

Pollen deposition on the stigma per bee visit was measured using emasculated (pollen-excluded) flowers. We haphazardly selected 51 flower buds in the field (Na-4, Na-5, Na-6, and Na-7) and bagged them until they opened. We then removed pollen grains attached to the lateral surface of the style in each newly opened flower (i.e., male phase) and then rebagged the flower. After the flower reached the female phase (stigma exposed), we removed the bag again to allow bumblebees to visit the flower. We timed each bumblebee visit. After a single visit, we caught the bumblebee, identified its species and caste, and measured its body size (PL and PH). We also measured the floral size (CE, CW, SL, and CL). Then, we collected the stigma cutting from the style, placed it on a slide, and sealed it with clear nail polish. We counted the pollen grains on the stigma under a light microscope (×100). The 51 flowers were visited by workers of *B. diversus* (*n* = 25) and workers (*n* = 23) and males (*n* = 3) of *B. beaticola*.

#### Effect of the flower–pollinator size match on pollen removal and pollen deposition

To determine the effect of the flower–pollinator size match on pollen removal and deposition, we estimated three size-match indices: entrance room (ER = CE − PH), style–pollinator room (SPR = [CW/2] − PH), and the ratio PL:SL (see Fig.2). If ER is too narrow, some bumblebees might not be able to enter the corolla. If SPR is too narrow, the bee might dislodge pollen, thus increasing the wasteful pollen fall, whereas if SPR is too wide, pollen removal by the bee might be reduced. The PL:SL ratio determines the position of the bee's thorax on the style. We collected these data from each visited flower and visiting bumblebee described in the previous subsections.

We used a multiple regression analysis to evaluate the factors influencing plant fitness (where pollen removal and pollen deposition are the correlates of male- and female fitness, respectively) and the cost of a size mismatch (pollen fall). In this analysis, the flower–pollinator size match (ER, SPR, and PL:SL) and visit duration were used as predictive variables after standardization (mean = 0, variance = 1). We also performed a quadratic regression of the flower–pollinator size-match parameters against plant fitness to detect the kind of effect (directional, stabilizing, or disruptive) (Alexandersson and Johnson 2002; Anderson and Johnson 2009). Both the linear coefficient (*β*) and the quadratic coefficient (*γ*) were examined for each size-match index. We then performed additional single linear or quadratic regression analyses for each variable found to significantly influence plant fitness in the multiple regression analysis. These analyses were performed with R version 2.15.0 software (R Development Core Team 2012).

## Results

### Variations in local pollinator assemblages

The bumblebee pollinator assemblages changed along the altitudinal gradient (Fig.4). A total of 1140 bumblebee visits to *C. punctata* patches were recorded. The flowers in high-altitude populations (1900–2300 m a.s.l.) were visited almost exclusively by the smallest bumblebee species, *B. beaticola* (mostly workers), whereas flowers in the low-altitude (700―1000 m a.s.l.) populations were mainly visited by medium-sized *B. ardens* (males) and large-sized *B. diversus* (workers). Flowers of middle-altitude (1400―1700 m a.s.l.) populations in general received mixed visitations of small *B. beaticola* and large *B. diversus*; exceptionally intensive visitation by the largest species, *B. consobrinus*, was recorded only for the Ut-2 population (1531 m a.s.l.). Overall, the pollinator size parameters (PH and PL) of the populations tended to diminish as altitude increased, except for the Ut-2 population (Fig.4, Pearson's correlation coefficient; PH vs. altitude, *r* = −0.83, *P* < 0.001; PL vs. altitude, *r* = −0.64, *P* < 0.01).

Although four bumblebee species dominated the assemblages, two additional bumblebee species, *B. honshuensis* and *B. ussuriensis*, were observed at low frequencies at low- to middle-altitude populations (Fig.4). Two other bumblebee species distributed in the study region, *B. terrestris* and *B. norvegics*, visited other flower species (e.g., *Cirsium* spp.), but did not visit *C. punctata* at all. Other insect visitors (small bees, ants, earwigs, and small beetles) were observed sporadically on *C. punctata* flowers. It is doubtful that they functioned as effective pollinators because of their small body size and their low visitation frequency.

### Floral size variation

**Figure 5:**
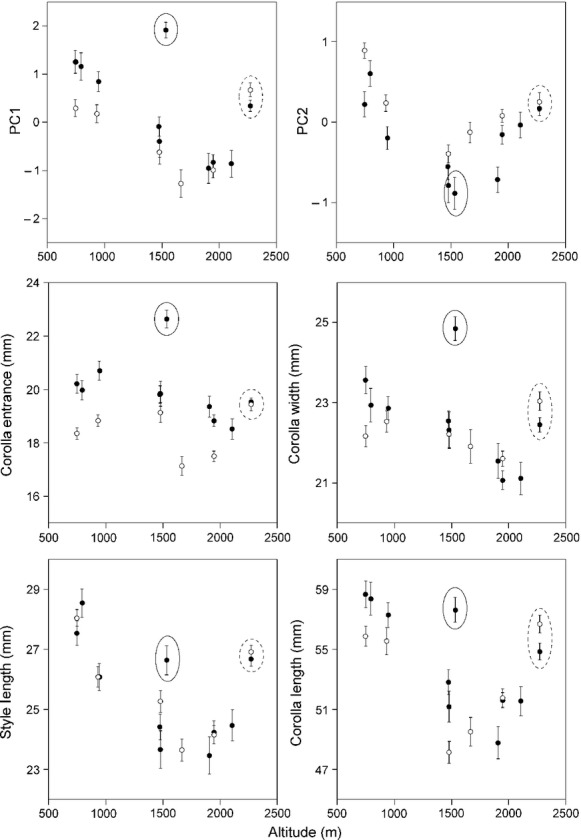
Altitudinal variation in the four floral size parameters (CE, CW, SL, and CL; see Fig.2) and the PC1 and PC2 scores (mean ± SE). Each circle represents a population (white, censused in 2010; black, censused in 2011). The PC1 score and the four size parameters tended to decrease with altitude. Outlier populations are circled: Ut-2, solid line; Na-7, dashed line.

**Figure 6:**
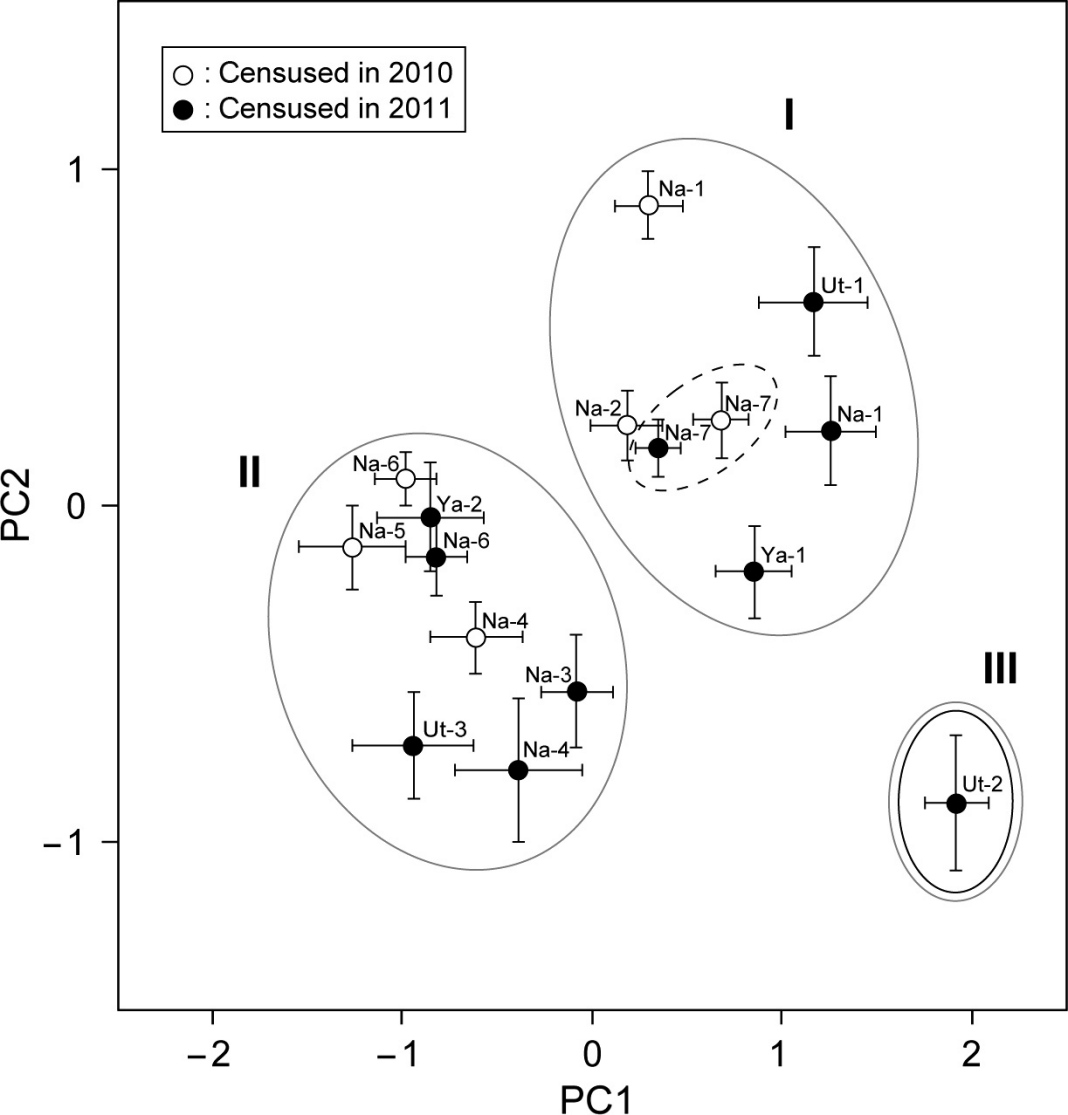
Scatter plots of PC1 scores versus PC2 scores (mean ± SE, estimated from the floral size parameters). Each symbol represents a population mean, and the bars show the standard errors (white, censused in 2010; black, censused in 2011). We identified three clusters: I, large-flower populations; II, small-flower populations; and III, population Ut-2, which had large, wide flowers (see Table2). Outlier populations are circled: Ut-2, solid line; Na-7, dashed line (see Fig.5).

We detected significant variations in the four floral size parameters (CE, CW, SL, and CL) among populations (CE, *F* = 18.65, *P* < 0.001; CW, *F* = 10.18, *P* < 0.001; SL, *F* = 24.6, *P* < 0.001; CL, *F* = 23.22, *P* < 0.001). Except for populations Ut-2 and Na-7, floral size tended to diminish as altitude increased (Fig.5). We also detected differences in PC1 and PC2 scores among populations (PC1, *F* = 22.84, *P* < 0.001; PC2, *F* = 17.73, *P* < 0.001); in particular, the PC1 scores tended to decrease as altitude increase, although populations Ut-2 and Na-7 were outliers (Fig.5). The number of flowers per plant (NFP) also differed among populations (*F* = 20.35, *P* < 0.001), with no apparent altitudinal trend (Pearson's correlation coefficient; *r* = -0.07, *P* = 0.17). In the PCA, PC1 accounted for 62% and PC2 for 23% of the total floral size variation (Table2). PC1 was positively correlated with all four floral size parameters (Table2). In scatter plots of PC1 versus PC2, we identified three clusters (I, II, and III in Fig.6). The flowers in populations of cluster I had larger corollas, those in cluster II populations had smaller corollas, and the flowers in population Ut-2 (cluster III) had large, wide corollas.

**Table 2:** Principal component analysis results. The relative contribution of each of the first two components, PC1 and PC2, to the total variance is shown, along with the factor loadings of the four flower size parameters of *Campanula punctata* on the two PC axes. Together, PC1 and PC2 account for 84.83% of the total variance.

	PC1	PC2
Contribution rate (%)	62.15	22.68
Corolla entrance (CE)	0.77	0.52
Corolla width (CW)	0.86	0.35
Style length (SL)	0.68	−0.64
Corolla length (CL)	0.83	−0.32

### Bee preference for floral size

We detected no floral size preference of *B. diversus* or *B. beaticola* in *C. punctata* populations (Na-4 and Na-5, corolla length [mm, mean ± SE]: flowers visited by *B. diversus*, 51.70 ± 0.96; flowers visited by *B. beaticola*, 49.72 ± 1.89; *t*-test, *P* = 0.312).

### Factors influencing local floral size

In the GLMM analysis, pollinator size (PH or PL) generally influenced flower size (Table3). PH positively affected PC2 and CW, and PL positively affected PC1, SL, and CL and negatively affected PC2. The number of flowers per plant positively affected CW and CL (Table3). Although the effect of population altitude on PC2 was positive, it had no effect on any of the four flower dimensions or PC1 (Table3).

**Table 3:** Outcome of the generalized linear mixed model (GLMM) analysis testing the effect of altitude, the number of flowers per plant, and pollinator size (PH and PL, see Fig.2) on the floral size of the population. Floral size was quantified as corolla entrance diameter (CE), corolla width (CW), style length (SL), corolla length (CL), and the PC1 and PC2 scores of each population.

Factor	PCA score				Flower width				Flower length			
		
	PC1		PC2		CE		CW		SL		CL	
					
	*β* (SE)	*t*	*β* (SE)	*t*	*β* (SE)	*t*	*β* (SE)	*t*	*β* (SE)	*t*	*β* (SE)	*t*
Altitude	0.001 (0.001)	1.940	**0.001 (0.000)**	4.247**	0.001 (0.001)	1.149	**0.002 (0.001)**	**2.097**^ms^	0.001 (0.001)	0.690	0.002 (0.002)	1.398
No. of flowers	0.024 (0.018)	1.303	0.002 (0.012)	0.168	−0.005 (0.026)	−0.197	**0.055 (0.027)**	2.049*	−0.031 (0.035)	−0.871	**0.153 (0.071)**	2.147*
PH	0.056 (1.213)	0.965	**3.374 (0.435)**	7.763***	2.143 (1.302)	1.646	**2.500 (0.951)**	2.630*	–		–	
PL	**0.366 (0.154)**	**2.378**^ms^	−**0.421 (0.057)**	−7.394***	–		–		**0.343 (0.171)**	2.495*	**0.852 (0.300)**	2.843*

Values in bold are significant:

*** *P* < 0.001;

** *P* < 0.01;

* *P* < 0.05;

ms, marginally significant (*P* < 0.1).

### Effect of the flower–pollinator size match on pollen removal and pollen deposition

**Figure 7:**
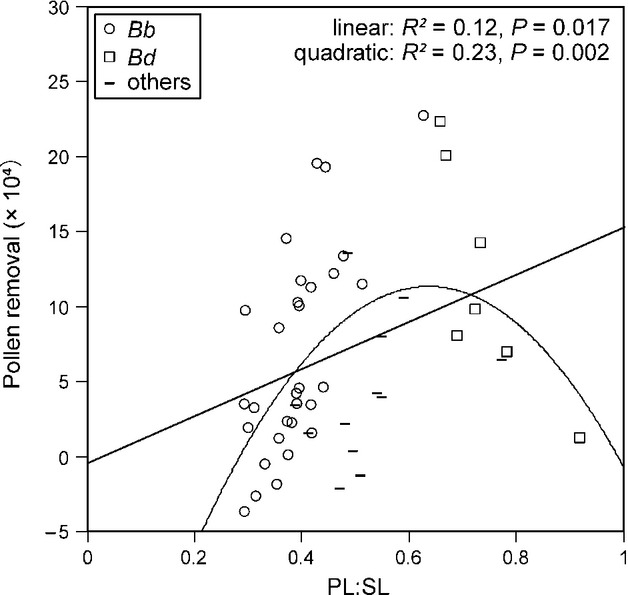
Male fitness (as indicated by pollen removal) as a function of PL:SL. Fitted single linear and quadratic regression lines are also shown. *Bb*, *B. beaticola* worker; *Bd*, *B. diversus* worker.

The size match between flowers and bumblebees affected male fitness of the plants, but not female fitness. Among the three size-match indices, only the PL:SL ratio affected plant fitness (male fitness = pollen removal by bees, *β* = 0.73, *P* = 0.03, *γ* = −0.32, *P* = 0.009, Table4). Single regression analyses revealed that PL:SL significantly affected male fitness: linear regression, *R*^2^ = 0.12, *P* = 0.017; quadratic regression, *R*^2^ = 0.23, *P* = 0.002 (Fig.7). PL:SL also affected pollen fall (*β* = 0.48, *P* = 0.030, Table4). Single linear regression analyses revealed that PL:SL significantly influenced pollen fall (*R*^2^ = 0.21, *P* < 0.001). Although the size match did not affect female fitness (pollen deposition on the stigma), the effect of visit duration was significant (*β* = 0.47, *P* < 0.001, Table4). Single linear regression analyses revealed that visit duration significantly influenced female fitness (*R*^2^ = 0.22, *P* < 0.001).

**Table 4:** Results of multiple regressions examining the effect of the size match between flowers and bumblebees (and pollinator visit duration) on plant fitness. Three indices of size matching were estimated: entrance room (ER) = CE (corolla entrance width) − PH (pollinator height); style–pollinator room (SPR) = (CW/2) − PH (CW, corolla width), and the ratio of pollinator length to style length (PL:SL) The dimension represented by each size parameter is illustrated in Fig.2.

Fitness	Index	Linear	Quadratic
		*β* (SE)	γ (SE)
Pollen removal from style (male fitness)	ER	−0.27 (0.22)	0.09 (0.11)
	SPR	0.24 (0.21)	−0.05 (0.14)
	PL:SL	0.73 (0.21)**	−0.32 (0.19)**
	Duration	0.02 (0.14)	
Pollen fall (cost of size-mismatch)	ER	0.15 (0.20)	−0.05 (0.11)
	SPR	−0.07 (0.21)	−0.09 (0.14)
	PL:SL	0.48 (0.22)*	−0.01 (0.12)
	Duration	−0.06 (0.14)	
Pollen deposition on stigma (female fitness)	ER	0.16 (0.20)	0.09 (0.12)
	SPR	−0.21 (0.19)	0.09 (0.12)
	PL:SL	−0.16 (0.14)	−0.01 (0.11)
	Duration	0.47 (0.13)***	

Values in bold are significant:

*** *P* < 0.001;

** *P* < 0.01;

* *P* < 0.05.

## Discussion

We found apparent geographic variations in assemblages of pollinators of *C. punctata* along an altitudinal gradient, one consequence of which was a geographic difference in pollinator size (Fig.4). The floral size of *C. punctata* also varied along an altitudinal gradient (Fig.5), but these variations reflected the local pollinator size; they were not strongly influenced by altitude itself (Table3). Although the results are correlative with no manipulative experiments, they are bolstered by the replication in 3 mountains. Furthermore, the size match between the flowers and the bees, as indicated by the PL:SL ratio, influenced plant male fitness (Table4). These results strongly suggest that *C. punctata* floral size is under pollinator-mediated selection and that the geographic mosaic of locally adapted *C. punctata* occurs at a fine spatial scale.

### Variations in local pollinator assemblages

At high altitude, the small bumblebee species, *B. beaticola* (worker), was the most frequent visitor at *C. punctata* flowers, whereas at low altitude, large *B. diversus* (worker) and medium-sized *B. ardens* (male) were the main visitors (Fig.4). This distribution pattern of the bumblebees largely reflects the altitudinal ranges of these species in central Japan (Kato et al. 1993; Tomono and Sota 1997). It is notable, however, that the assemblages of bumblebees visiting *C. punctata* flowers in the same altitudinal range differed among populations. For example, although the largest bumblebee species, *B. consobrinus* (worker), was the main visitor at Ut-2 (1531 m a.s.l.), the main visitors of *C. punctata* flowers at Na-3, Na-4, and Na-5 (1400–1700 m a.s.l.) belonged to different bumblebee species, even though the four populations were at the same altitudinal range (Table1, Fig.4). This geographically variable pattern of bumblebee visitation to *C. punctata* may be influenced by the location of the bumblebee nests. For instance, Suzuki et al. (2007, 2009) suggested that the nesting location of bumblebees is influenced by the floral resource distribution during the nest-initiating period of bumblebee queens.

These facts suggest that the bumblebee assemblages visiting *C. punctata* at different places, which may be affected by either abiotic clinal environmental changes along altitude and/or biotic geographic changes (e.g., relative abundance of flowering plants), may exert geographically different selective pressures on the floral size of *C. punctata*.

### Factors influencing local floral size

We showed that the local floral size of *C. punctata* was influenced by the average size of local pollinators (Table3). Other factors known to affect local floral size include abiotic factors due to meteorological differences along altitude (Galen 1999; Strauss and Whittall 2006). For instance, stress caused by draught can reduce floral size (Galen 1999; Lambrecht and Dawson 2007; Teixido 2013). Many environmental changes show a clinal pattern along altitude (Körner 2007). However, in our GLMM analysis, we found no effect of altitude itself on floral size parameters, except on PC2 (Table3). Therefore, changes in abiotic factors along altitude apparently do not strongly affect floral size in *C. punctata*.

One factor that might affect floral size is the soil nutrient status. Vogler et al. (1999) reported that, in *Campanula rapunculoides*, the number of flowers per plant correlates with soil nutrient conditions, and in *C. punctata* as well, the number of flowers per plant increases dramatically in plants growing where soil nutrient conditions are favorable (Y. Nagano, personal observation). If floral size is affected by the soil at a site, then floral size should correlate with the number of flowers per plant at that site. Or, if there is a trade-off between the number of flowers and the floral size, as has often been observed in plants (Bell 1985; Sakai 2000), then the number of flowers and floral size should be negatively correlated. Contrary to these predictions, we found no effect of the number of flowers per plant on CE or SL, but flower number did show a significant positive correlation with CW and CL (Table3). This result suggests that soil nutrients or a trade-off between the number of flowers and flower size cannot fully explain the floral size variation.

Another factor that may influence floral size is selection pressure from herbivores (Strauss 1997; Breadmore and Kirk 1998). In this study, however, no organisms were observed to cause severe damage to flowers and leaves of *C. punctata*. Although nectar robbers can also potentially influence floral size (reviewed by Irwin et al. 2010), we observed no nectar robbers of *C. punctata* except for a few very small insects (ants or earwigs).

The correlation between local floral size of *C. punctata* and the average size of local pollinators (Table3) can be attributed to at least two possible mechanisms. An adaptive floral size may be selected for by the local bee size (bees-came-first hypothesis), or local floral size may vary because of unknown factors and each bumblebee species may preferentially visit right-sized *C. punctata* (flowers-came-first hypothesis). If the flowers-came-first hypothesis is correct, then the larger bees such as *B. diversus* should prefer larger *C. punctata* flowers and smaller bees such as *B. beaticola* should prefer smaller flowers in the same *C. punctata* population. However, we detected no flower size preference of *B. diversus* or *B. beaticola* in populations of *C. punctata* visited by both bumblebees. This result suggests that the floral size of *C. punctata* does not influence the flower visitor assemblage; rather, the difference in bumblebee assemblages among populations might influence *C. punctata* floral size.

Exceptionally, at Na-7, floral size was relatively large in comparison with bumblebee size (Figs.4 and 5). Nattero et al. (2010) reported that phenotypic matching is not necessarily an expected outcome in a specialized pollination system and proposed that temporal fluctuation in the pollinator assemblage might cause a mismatch. Although the pollinator assemblages did not differ between the two study years, 2010 and 2011, at the Na-4, Na-6, and Na-7 populations (Fig.5), pollinator assemblages might fluctuate over longer periods. It is possible that the large floral size at Na-7 may have been selected for in the past by a larger *Bombus* species than *B. beaticola*, such as *B. consobrinus*.

### Effect of the flower–pollinator size match on pollen removal and pollen deposition

Pollen removal from the flower style onto bees (male fitness) was strongly influenced by the size match between style length and pollinator length (i.e., PL:SL) (Table4). Because the coefficient of determination in single quadratic regression (*R*^2^) was higher than in single linear regression (Fig.7), a stabilizing selection-like effect appears to act on style length of *C. punctata*. To collect the flower nectar, the bumblebee inserts its galea, which is a relatively solid organ, into the cover that conceals the nectar space (Fig.2). While the bee is collecting nectar, pollen grains stick to the dorsal side of its thorax. Thus, the PL:SL ratio indicates the position of the bee's thorax on the style while the bee is collecting nectar. Because most grains adhere to the middle part of the style (about one-third of the distance from the tip), the optimal PL:SL should be around 2:3. In fact, when PL:SL was larger or smaller than this value, pollen removal by the bumblebees decreased (Fig.7). This result (Fig.7) suggests that *B. diversus* is too big for the floral size of the investigated populations and *B. beaticola* is too small. In the four investigated populations (Na-4, Na-5, Na-6, and Na-7) that were visited by both *B. diversus* and *B. beaticola* (Fig.4), the observed style length did not fit the body size of either species, but appeared to be intermediate (Fig.7).

Kobayashi et al. (1997, 1999) suggested that variation in corolla width (CW) influences pollen fall in *C. punctata* (and *C. microdonta*). On the other hand, they showed that corolla length (CL) does not influence plant male fitness. In this study, neither of the corolla width parameters (CE, CW) nor CL affected plant fitness, although style length (SL) did. Possibly, corolla size (CE, CW, and CL) affect plant fitness through pollinator attraction during the first step of the pollination process (Bell 1985; Johnson et al. 1995; Conner and Rush 1996), instead of through pollen removal or deposition. Additionally, the unnatural experimental setting of Kobayashi et al. (1997, 1999) appeared to flaw the results. They used *C. microdonta* which is distributed in bumblebee-free islands in Japan and has very small-sized flowers adapted to small bee pollinators. They potted and brought *C. microdonta* to mainland Japan, and offered it to the large-sized bumblebee (*B. diversus*). Such artificial combination of the flower and the bee may have resulted in the observed tremendous pollen fall in their experiments. While Kobayashi et al. (1997, 1999) did not measure style length, our study clearly showed that pollination efficiency in wild *C. punctata* is influenced by the size match between style length and bumblebee size.

Pollen deposition on the stigma (female fitness) was strongly influenced by the bumblebee visit duration (Table4). We observed that when bumblebees visited the flowers, they moved around in the corolla and shifted their position in order to insert their galea into the nectar space. Therefore, the prolongation of such foraging behavior can be expected to increase pollen deposition on the stigma. Based on the assumption that *C. punctata* is pollen-limited, we measured pollen deposition on stigma as a proxy for female fitness. However, as Kobayashi et al. (1997) suggested that seed set in *C. punctata* is not pollen-limited, future research is required to count fruit set – and ideally seed viability as a measure of female fitness. Furthermore, male fitness would preferably be measured as the number of offsprings produced, rather than the pollen removal following one visit from a single pollinator.

Is the geographic differentiation in floral traits of *C. punctata* driven by spatially variable selection by pollinators? We answer this question in terms of the five steps needed to be satisfied to demonstrate geographic floral adaptation driven by selection from pollinators (Herrera et al. 2006). (1) Did we document geographic variation in pollinator assemblages? Yes. In *C. punctata*, pollinator assemblages changed along altitude (Fig.4). (2) Did we demonstrate pollinator-mediated selection of floral traits? Yes. In *C. punctata*, the size match between flowers and bumblebees affect male plant fitness (Table4, Figs.5 and 7). (3) Did we observe geographic divergence in selection? Yes. In *C. punctata,* pollinator size, which poses selection pressure, changed geographically (Table4, Figs.4, 5, and 7). (4) Did we find a match between divergent selection and phenotypic divergence? Yes. In *C. punctata,* pollinator size and floral size were geographically correlated (Table3). (5) Do the observed population differences in floral traits have a genetic basis? The genetic basis of the floral traits was not assessed in this study, but Inoue et al. (1995) showed that floral size in *C. punctata* var. *punctata* has a genetic basis. In conclusion, our results strongly suggest that floral size in the herb *C. punctata* is locally adapted to the local pollinator size at a very fine spatial scale in a steep and diverse mountain environment.
